# Variable Baseline *Papio cynocephalus* Endogenous Retrovirus (PcEV) Expression Is Upregulated in Acutely SIV-Infected Macaques and Correlated to STAT1 Expression in the Spleen

**DOI:** 10.3389/fimmu.2019.00901

**Published:** 2019-05-15

**Authors:** Emmanuel Atangana Maze, Claire Ham, Jack Kelly, Lindsay Ussher, Neil Almond, Greg J. Towers, Neil Berry, Robert Belshaw

**Affiliations:** ^1^School of Biomedical Sciences, Faculty of Medicine and Dentistry, University of Plymouth, Plymouth, United Kingdom; ^2^Division of Infectious Disease Diagnostics, National Institute of Standards and Control (NIBSC), Potters Bar, United Kingdom; ^3^Division of Infection and Immunity, University College London, London, United Kingdom

**Keywords:** endogenous retrovirus, STAT1, macaque, PcEV, SIV, RNA, innate immunity

## Abstract

Retroviral replication leaves a DNA copy in the host cell chromosome, which over millions of years of infection of germline cells has led to 5% of the human genome sequence being comprised of endogenous retroviruses (ERVs), distributed throughout an estimated 100,000 loci. Over time these loci have accrued mutations such as premature stop codons that prevent continued replication. However, many loci remain both transcriptionally and translationally active and ERVs have been implicated in interacting with the host immune system. Using archived plasma and tissue samples from past macaque studies, experimentally infected with simian immunodeficiency virus (SIV), the expression of one macaque ERV in response to acute viral infection was explored together with a measure of the innate immune response. Specifically, RNA levels were determined for (a) *Papio cynocephalus* Endogenous Retrovirus (PcEV), an ERV (b) STAT1, a key gene in the interferon signaling pathway, and (c) SIV, an exogenous pathogen. Bioinformatic analysis of DNA sequences of the PcEV loci within the macaque reference genome revealed the presence of open reading frames (ORFs) consistent with potential protein expression but not ERV replication. Quantitative RT-PCR analysis of DNase-treated RNA extracts from plasma derived from acute SIV-infection detected PcEV RNA at low levels in 7 of 22 macaques. PcEV RNA levels were significantly elevated in PBMC and spleen samples recovered during acute SIV infection, but not in the thymus and lymph nodes. A strong positive correlation was identified between PcEV and STAT1 RNA levels in spleen samples recovered from SIV-positive macaques. One possibility is that SIV infection induces PcEV expression in infected lymphoid tissue that contributes to induction of an antiviral response.

## Introduction

Endogenous Retroviruses (ERV) are descendants of ancient retroviral infections that have become established in the germline and proliferated to now represent ~5% of the human genome and other mammals, rising to ~8% if the older group of Mammalian Apparent LTR-Retrotransposons (MaLRs) are included. Individual proviruses, termed loci, accumulate mutations over time during their Mendelian transmission that eventually render them replication-defective. In the human reference genome, no locus has retained full-length Open Reading Frames (ORFs) for all genes which precludes the possibility of yielding infectious, cell-free progeny virions (1, 2). However, ERVs and other retroelements have been shown to play a role in epigenetic gene regulation (3–7), with many ERV loci having been co-opted. Examples include HERV-E Long Terminal Repeat (LTR)-driven tissue-specific expression of a human salivary amylase gene (8), ERV-derived syncytins that play a role in placentation (9), and promoter-containing LTRs of ERVs that influence epigenetic gene control and pluripotency (10).

The study of ERVs in non-human primates (NHP) has potential to broaden the wealth of data generated from mouse studies. Smaller mammals tend to have more ERV loci integrating into their genome than larger mammals (11) and, consistent with this general pattern, replication-competent ERV loci have been found in the mouse (12) but not in humans (1, 2, 12–14). In terms of the number of recently integrated ERV loci, the macaque genome is more similar to the human than the mouse (11). Macaques thus represent an opportunity to derive additional relevant data that could augment more conventional mouse studies. To help bridge this gap we have utilized archived materials derived from previous simian immunodeficiency virus (SIV) studies.

Recently, it has been suggested ERVs and other retroelements play a role in innate immune signaling by acting as Pathogen Associated Molecular Patterns (PAMPs) (15–21). Indeed, it has been proposed that retroelement PAMPs, such as cytosolic DNA resulting from reverse transcription, set an activation threshold for triggering the innate immune response (22). These authors speculate that the level at which activation occurs is an evolutionary trade-off between avoidance of tolerance of exogenous pathogens on one hand and constant triggering by endogenous elements on the other. The latter appears to occur in the inherited autoimmune disease Aicardi-Goutieres syndrome where deficiency of TREX1 DNA endonuclease may lead to a build-up of retroelement PAMPS, which triggers inappropriate innate signaling (23). If ERVs were involved in the early immune response, it might be expected that a correlation would exist between ERV expression and measures of innate immune activation in archived macaque samples taken during acute infection.

Our study focuses on *Papio cynocephalus* Endogenous Retrovirus (PcEV), a recently integrated ERV first described in baboons (24). PcEV is one of three ERV lineages copying within the macaque genome during the last 5 million years (25). Since a range of components in the ERV replication cycle can potentially be detected by the innate immune system (26), we argue that the likelihood of an ERV locus being biologically relevant increases if its sequence is free of inactivating mutations such as premature stop codons. Induction of type-1 interferons (IFN-1), via the JAK/STAT pathway, represents a key part of the innate response to viral infections (27, 28). Interferon-stimulated genes (ISGs) act to limit viral replication and play a role in the control of the viral burden (29). However, the immunomodulatory role of ISG induction in acute-phase and chronic SIV infection is complex and relatively poorly understood, contributing to both dampening and inflammatory immune responses (30, 31). IFN-1 signaling results in the transcription of hundreds of ISGs, an important member being STAT1 (32, 33). Basal IFN-1 levels set the abundance of STAT1, which influences the susceptibility toward infection (34). STAT1 therefore provides a measure of IFN-1 activation induced during acute SIV infection in macaques (35), hence its expression was explored in this study.

Archived macaque plasma and tissue samples were analyzed to determine PcEV activity during acute SIV infection when *de novo* responses are likely to be highest, particularly lymphoid tissues targeted during primary infection (35). This activity was also compared with STAT1 RNA levels in the same RNA preparations. Our study represents the first analysis of PcEV loci in the macaque reference genome sequences and demonstrates the presence of multiple potentially protein-coding, but not replicating, PcEV loci. In conjunction with identification of low levels of PcEV RNA in plasma of a proportion of acutely SIV-infected macaques, PcEV was actively transcribed in tissues with a level of cell-associated gene expression that is upregulated in response to acute SIV infection. In the spleen, this appeared to be directly correlated to STAT1 RNA expression. The potential significance of these findings linking ERV and innate immunity are discussed.

## Results

### Bioinformatic Analysis Suggests PcEV Protein Production but Not Replication

**Figure 1:**
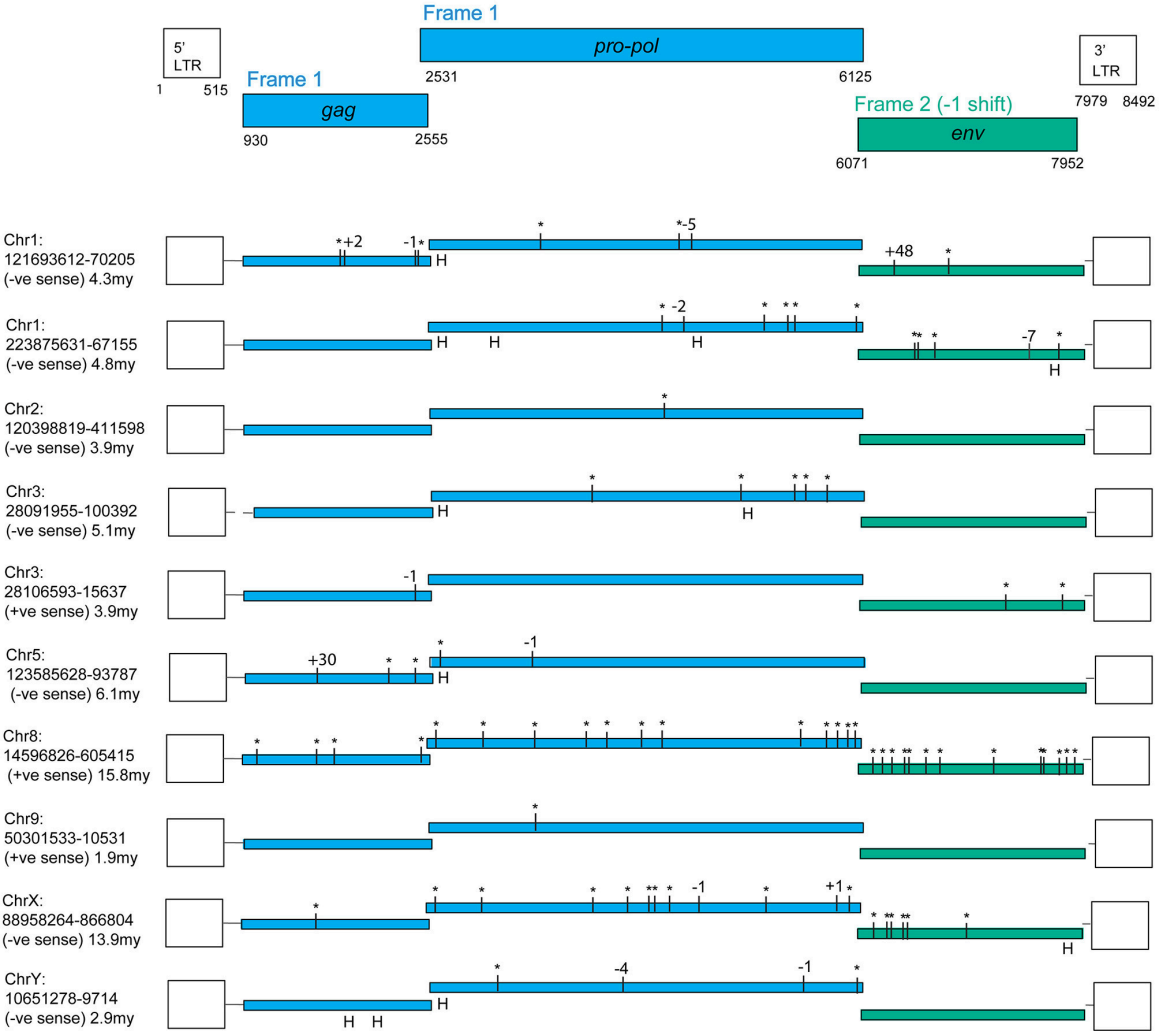
PcEV loci with full-length ORFs for some but not all genes. Premature stop codons are shown as asterisks and frame-shifting (or large in-frame) indels are shown by the number of nucleotides involved (“H” marks an indel within a homopolymer, assumed to be sequencing errors). Results are from the rheMac8 build with all interruptions either confirmed or corrected using the earlier rheMac2 build from the same animal, i.e., interruptions were treated as sequencing errors if present only in one build. Details of assembly problems in these loci are in Supplementary Information, which also contains multiple alignments for the three genes, the LTR alignments used for dating, the flanking regions used to determine locus homology across genomes, and reference (consensus) sequences both for the complete provirus and individual genes. *Pro-pol* alignment starts from the position suggested in Mang et al. (24). The reference coordinates here include a one nucleotide gap inserted at the end of *pro-pol* to incorporate the frameshift in *env*. Note, the *gag* and *pro-pol* are translated in the same frame with suppression of the *gag* stop codon (36).

An online search of the most recent rhesus macaque (*Macaca mulatta*, RM) genome assembly (rheMac8) using the constructed PcEV reference sequence revealed 72 PcEV loci (plus another 76 matches in unassembled regions), most of which are represented only by fragments. Figure 1 illustrates the only 10 loci that do not have large regions (more than a few hundred nucleotides) missing from the reference genome. Among these loci are examples of full-length ORFs for all genes but no single locus has full-length ORFs in all genes.

The fragmentation observed is largely the result of genome assembly errors. Four of these 10 most intact loci contain scaffold gaps, where sequenced regions either side have not been joined. One example is locus chr9:50301533-10531, which in the rheMac8 assembly has its *pro-pol* reading frame interrupted by a single premature stop codon and by a long insertion which consists of a tandem duplication at a region marked as a scaffold gap. However, an examination of this locus sequence from an earlier assembly of the RM genome (rheMac2), which was from the same individual animal, and from the homologous locus in the cynomolgus macaque (*Macaca fascicularis*, CM) genome (macFas5), a species that diverged from the RM ~1–2 million years ago (mya) (37, 38), demonstrates that neither the premature stop codon nor the insertion belong to this locus. These three *pro-pol* sequences are not homologous along their entire length and have a clearly visible transition, a “breakpoint,” near to position 3,937 in the reference genome: the ~40% of the rheMac8 *pro-pol* sequence before this breakpoint is no more similar to the other two sequences than it is to a range of other PcEV loci, while downstream the sequences of all three loci are very similar.

**Figure 2:**
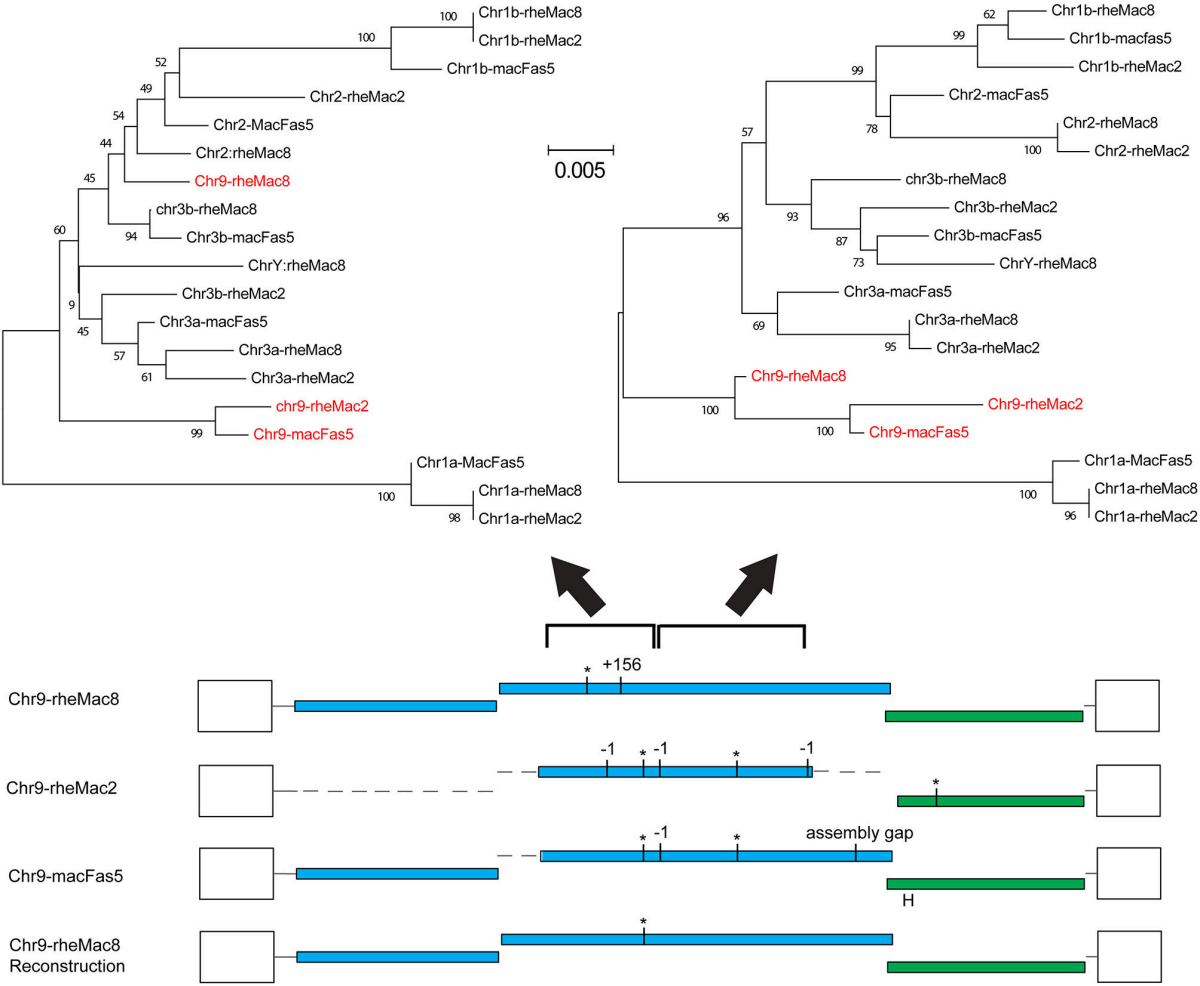
Example of assembly error in reference genome. Reconstruction of the locus chr9:50301533-10531 using homologous sequences in the 2 RM genome assemblies (rheMac8 and rheMac2) and in the CM genome (macFas5). See Figure 1 legend for annotation of ORFs. Loci shown are those in Figure 1 (a/b = locus on chromosome with lower/higher coordinate value). Phylogenetic trees were built using NJ in MEGA from the 501 to 1,397 and the 1,398 to 2,607 regions of the pro-pol alignment, with sequences from this locus shown in red. Values below branches are bootstrap score (1,000 replicates).

This can also be demonstrated phylogenetically (Figure 2), where a tree of *pro-pol* sequences before the breakpoint shows the rheMac8 sequence is not recovered in the same clade as the rheMac2 and macFas5 sequences, while a tree of the *pro-pol* sequences after this break-point shows all three sequences recovered together in a well-supported clade (as seen in the other loci). This represents a clear assembly error. Indeed, the locus in rheMac2 and in macFas5 are in the antisense direction while the locus in rheMac8 is in the sense direction. Consistent with widespread assembly problems, only approximately half of the 'intact' loci in rheMac8 are intact in macFas5, and *vice versa*. There is also much more fragmentation in PcEV loci than in the human HERV-K(HML2) loci (39), which belong to an older ERV lineage (40). The human ERVs were only sequenced after being cloned within bacterial artificial chromosomes (41), which avoided the problem with the macaque genomes of trying to assemble simultaneously multiple very similar ERV loci from short next generation sequencing reads.

Evidence was identified of insertionally polymorphic PcEV loci, i.e., loci present in some individuals but not others (42). The intact loci recovered are mostly several millions of years old (Figure 1), the most recently integrated locus being chr9:50301533-10531. The LTRs of this locus differ by two substitutions which, given an estimated rate of nucleotide substitution of ~1 × 10^−9^/nucleotide/year (25), indicates an age of ~1.9 million years. All these loci are also found in the CM reference genome and are therefore expected to be fixed in the RM population. However, two loci with large scaffold gaps were estimated to have integrated within at least the last 700,000 years; both were insertionally polymorphic. Locus chr1:55452680-60247 is heterozygous for presence/absence (= provirus/pre-integration site) in the reference genome and locus chr5:114918771-21894 is represented solely by the pre-integration site in the rheMac3 genome sequence, which is from a different individual (from China rather than India; further details of these loci are included in Supplementary Information).

We infer from these observations that PcEV transcription arising from some of these 10 loci may result in PcEV protein production but is unlikely to result in PcEV replication.

### PcEV Loci Are Likely to be Transcribed and Lack STAT1 Binding Sites

**Figure 3:**
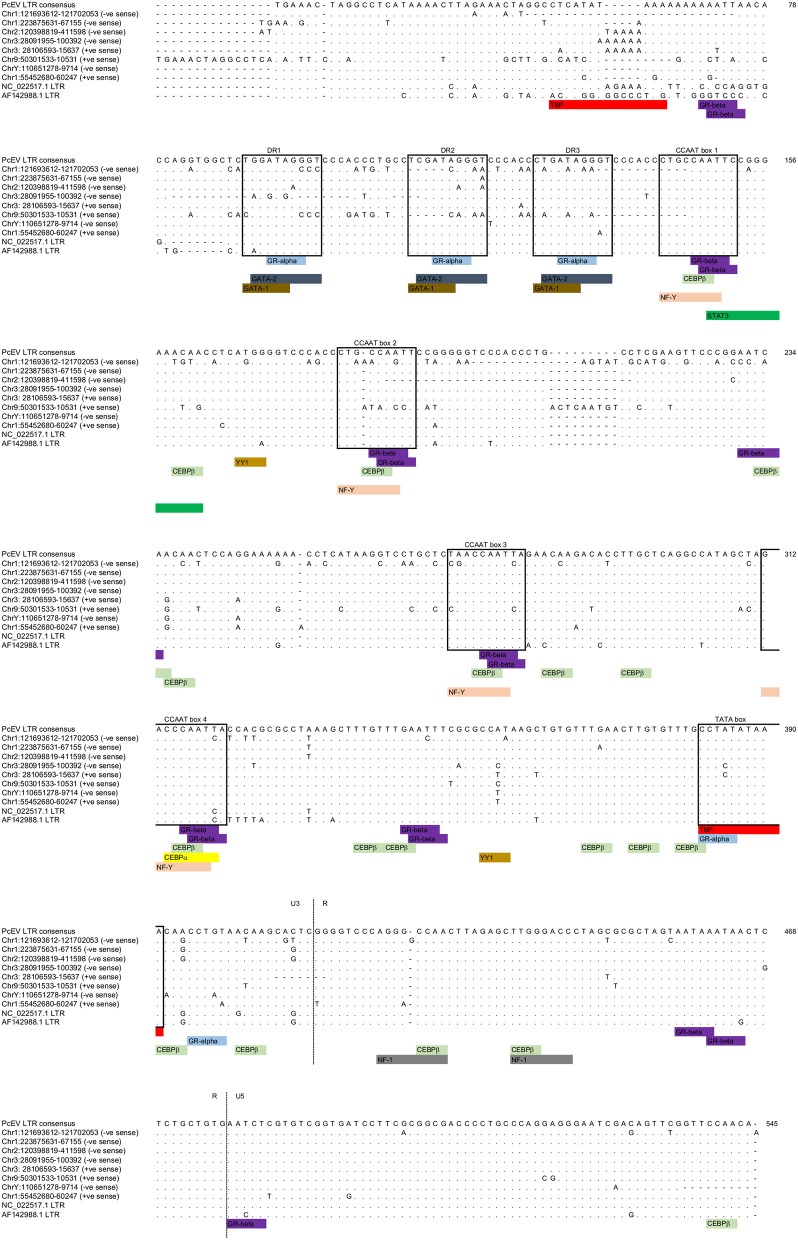
PcEV LTR TATA box and transcription factor binding sites. Prediction of transcription factor binding sites and TATA box in the eight PcEV loci described in the macaque genome in this study. LTR sequences from PcEV loci in the baboon (accessions NC_022517 and AF142988) are also shown.

The LTRs of the above loci, excluding the two very old ones, all have an undisrupted TATA binding protein (TBP) binding site (= TATA box) at position 382 (Figure 3; Table S1). This correlates to the position of the conserved TATA box found in PcEV in the baboon genome (24). Since the TATA box usually has at least minimal promoting activity (43), loci without disruption in the TBP binding site are likely to be transcribed. Another TBP binding site was predicted at position 51 but is unlikely to be real since it is poorly conserved among the loci and not found in other γ-retroviruses, such as HERV-W and MuLV (43, 44). The LTRs also contain many undisrupted transcription factor binding sites. The regions containing these sites correlated with ones already described in the baboon (24), so were named in accordance with that earlier study. Direct-repeat enhancers (DR1, DR2, DR3) contained sites for GRα, GATA1, and GATA2. Three CCAAT box-associated binding regions (CCAAT box 1, 2, and 3) contained sites for GRβ, CEBPβ, and NFY. A fourth CCAAT box region (box 4) contains these three sites plus the site for CEBPα. Such CCAAT boxes directly upstream of a TATA box have been shown to efficiently promote transcription activity of the HERV-W LTR (43). Other retroviral LTRs similarly harbor binding sites for many transcription factors (45).

The screening of PcEV loci for putative transcription factor binding sites included key inflammation-related transcription factors: STAT1, NFκB (46), and STAT3 (47). Predicted binding sites for STAT1 and NFκB were not identified, so it is therefore unlikely that STAT1 or NFκB directly regulates PcEV expression through binding to the retroviral promoter. The only inflammation-related transcription factor binding site found was for STAT3 at position 149 (Figure 3).

### Low Levels of Plasma PcEV RNA in One-Third of Acutely SIV-infected Macaques

**Figure 4:**
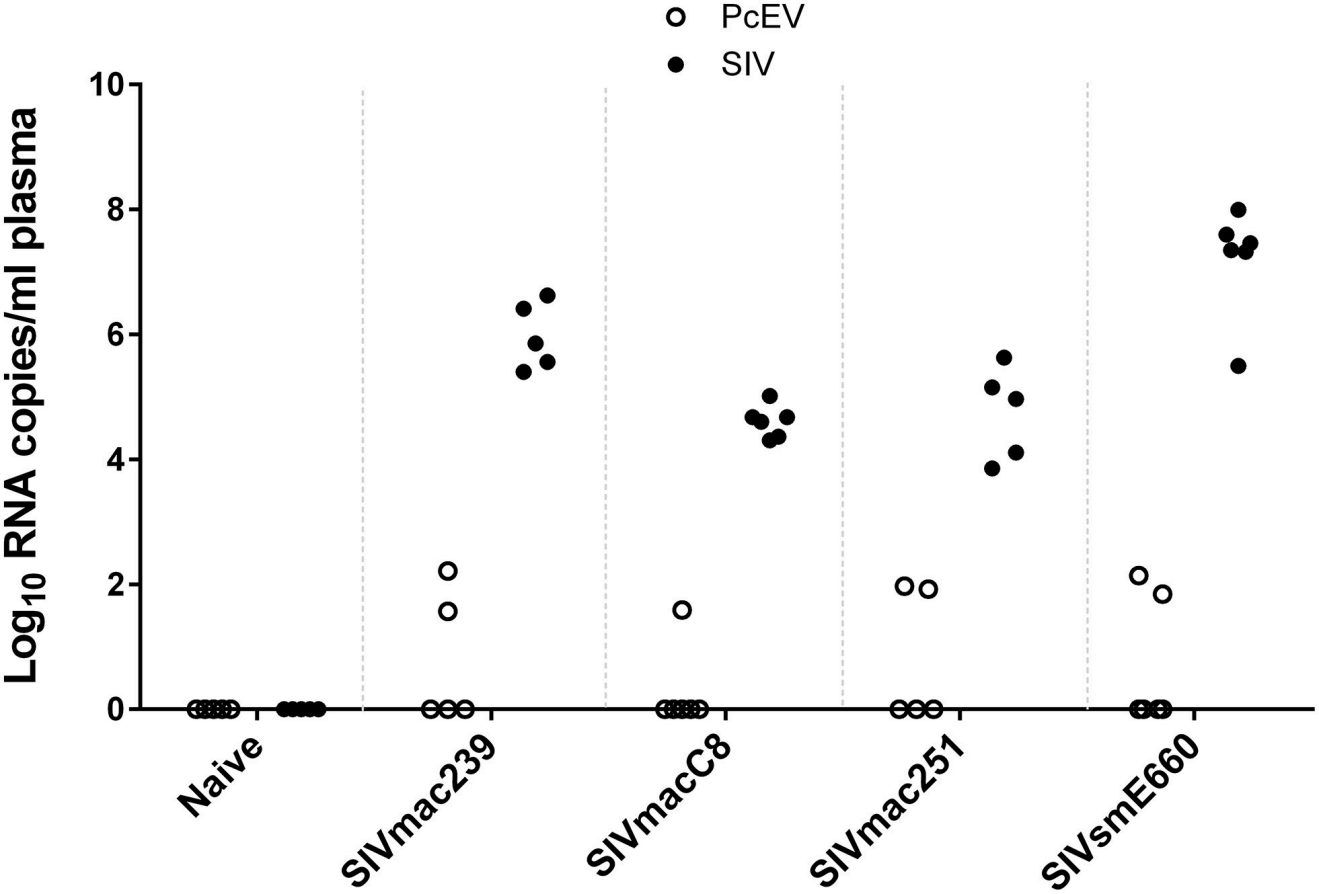
Low levels of PcEV RNA are present in the plasma of some SIV+ macaques. Viral RNA copies/ml plasma RNA in naïve and SIV+ macaques: SIVmac239 (48), SIVmacC8 (35), SIVmac251 (49), and SIVsmE660 (50). Absence of DNA contamination was confirmed in all experiments by simultaneous RT-qPCR without the RT enzyme and only results from the corresponding RT+ reactions are shown. Each symbol represents the number of RNA copies from a single individual; open circles denote PcEV, closed circles SIV.

Cell-free PcEV RNA levels were measured by RT-qPCR in plasmas from unchallenged control (naïve) macaques (*n* = 5) or macaques at or around peak SIV viraemia (SIV+) challenged with one of four different SIV strains; SIVmac239 (*n* = 5), SIVmac251 (*n* = 5), SIVmacC8 (*n* = 6), or SIVsmE660 (*n* = 6). SIVmacC8 represents a minimally attenuated *nef*-disrupted variant of wild-type SIVmac251/32H (35), while all others are wild-type SIVs belonging to the SIVmac/SIVsm lineage. All viral challenges were performed in CM, except for SIVmac239 conducted in RM. Three naïve animals were CM, two were RM. As shown in Figure 4, 7 of 22 macaques (32%) exhibited low but detectable levels of PcEV in their plasma (39; 84; 94; 139; 70; 35; 165 PcEV copies/ml). These seven macaques include instances of infection with each of the four SIV strains. PcEV RNA was not detected in the plasma of naïve macaques, although the small sample size does not enable any inference about the relationship between PcEV detection in plasma and SIV status (Fisher's Exact Test comparing presence vs. absence of PcEV in plasma, from naïve and pooled SIV+ macaques, p = 0.27). Stringent protocols were employed for DNase treatment to remove contaminating genomic DNA, including paired +/– RT reaction steps in each experiment (Figure S1). Neither PcEV nor SIV were amplified in RT minus reactions (Table 1), providing additional confidence that all PcEV molecules originated from an RNA template. Hence, low but detectable PcEV RNA was detectable in around one-third of SIV+ plasmas during acute infection.

**Table 1:** PcEV and SIV RNA levels in plasma as determined by qPCR with (+) and without (–) reverse transcriptase (RT).

		**PcEV RNA copies/ml**		**SIV RNA copies/ml**	
**SIV strain**	**Individual**	**RT+**	**RT-**	**RT+**	**RT-**
Naïve^a^	E79	No Ct	No Ct	No Ct	No Ct
	E80	No Ct	No Ct	No Ct	No Ct
	E81	No Ct	No Ct	No Ct	No Ct
SIVmac239^a^	E79	No Ct	No Ct	2.60 × 10^6^	No Ct
	E80	No Ct	No Ct	7.24 × 10^5^	No Ct
	E81	No Ct	No Ct	4.23 × 10^6^	No Ct
SIVmacC8^a^	E5	No Ct	No Ct	1.04 × 10^5^	No Ct
	E6	No Ct	No Ct	2.03 × 10^4^	No Ct
	G3	39	No Ct	4.01 × 10^4^	No Ct
	G4	No Ct	No Ct	4.75 × 10^4^	No Ct
	G5	No Ct	No Ct	5.14 × 10^3^	No Ct
	G6	No Ct	No Ct	2.31 × 10^4^	No Ct
SIVmac251^a^	J41	No Ct	No Ct	1.42 × 10^5^	No Ct
	J42	No Ct	No Ct	9.35 × 10^4^	No Ct
	J44	84	No Ct	4.29 × 10^5^	No Ct
	J17	No Ct	No Ct	7.22 × 10^3^	No Ct
	J18	94	No Ct	1.29 × 10^4^	No Ct
SIVsmE660^a^	G25	No Ct	No Ct	2.25 × 10^7^	No Ct
	G26	139	No Ct	3.13 × 10^5^	No Ct
	G27	No Ct	No Ct	2.12 × 10^7^	No Ct
	G28	No Ct	No Ct	3.97 × 10^7^	No Ct
	G7	70	No Ct	9.91 × 10^7^	No Ct
	G8	No Ct	No Ct	2.89 × 10^7^	No Ct
Naïve^b^	H17	No Ct	No Ct	No Ct	No Ct
	H18	No Ct	No Ct	No Ct	No Ct
SIVmac239^b^	H19	37	No Ct	3.64 × 10^5^	No Ct
	H20	165	No Ct	2.52 × 10^5^	No Ct

Species of macaque is indicated; CM, cynomolgus macaque; RM, rhesus macaque. PcEV RNA levels are expressed as simple integers; SIV RNA levels use a power of ten notation. Ct, cycle threshold.

a In CM.

b *In RM*.

### Variable Baseline Tissue PcEV Transcriptional Activity Is Upregulated During Acute SIV Infection

**Figure 5:**
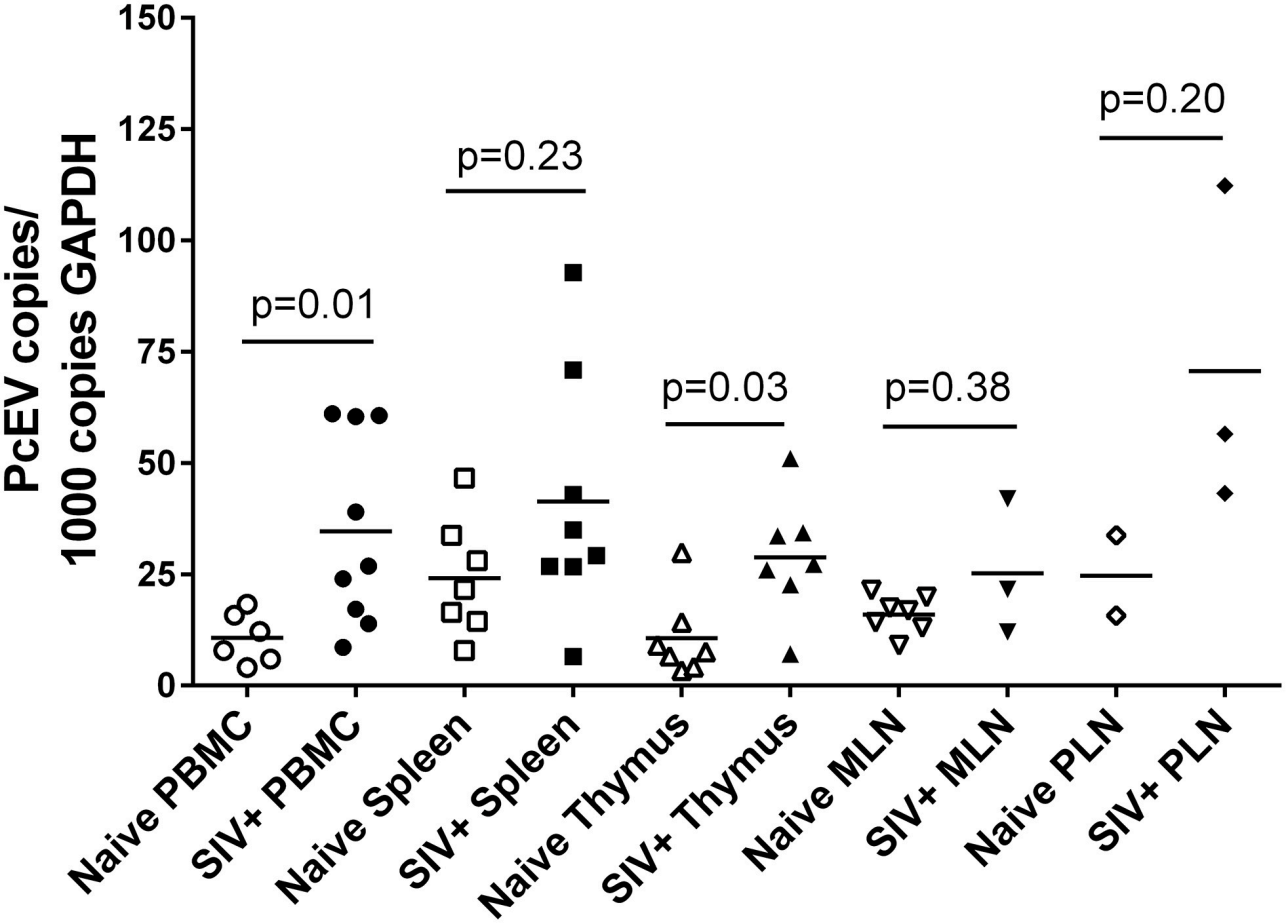
SIV infection upregulates PcEV expression. Comparison of PcEV expression in PBMC, spleen, thymus, MLN, and PLN. Open symbols represent naïve macaques and closed symbols represent SIV+ macaques. Each symbol represents the number of PcEV RNA copies from a single tissue from a single individual. Means for each group are indicated as horizontal bars with *p*-values given from a Mann-Whitney comparison of the ranked expression levels between naïve and SIV+ macaques.

Levels of detectable cellular PcEV transcripts in a range of tissues targeted by SIV were determined, isolating RNA from PBMC, spleen, thymus, peripheral and mesenteric lymph nodes (PLN/MLN) from naïve and acutely SIV+ macaques. Data generated for cell-associated PcEV transcriptional activity were compared for each preparation, normalized by co-amplification of the constitutively expressed cellular GAPDH gene with PcEV. Baseline PcEV RNA transcriptional activity was identified in the majority of tissues from all naïve macaques (Figure 5), though many were at low levels and none exceeded 60 PcEV RNA copies/1,000 copies GAPDH. In these naïve tissues, basal PcEV transcriptional activity for MLN, thymus, PBMC, spleen and PLN was determined as 19, 20, 25, 33, and 52 PcEV RNA copies/ml, respectively. Differences between these tissue means were not statistically significant (Kruskal-Wallis test, *p* = 0.07).

Mean PcEV levels were higher in tissues recovered from SIV+ macaques during acute infection (10–14 days post-infection) compared to naïve macaques (Figure 5). PcEV RNA was significantly increased in PBMC (Mann-Whitney test, *p* = 0.01) and thymus (*p* = 0.03), but increases were not significantly different in spleen (*p* = 0.23), MLN (*p* = 0.38), and PLN (*p* = 0.20). Differences between tissue means were not statistically significant (Kruskal-Wallis test, *p* = 0.83). Taken together, the data indicate that SIV infection *per se* upregulates the expression of PcEV RNA from a variable baseline level of transcriptional activity in a range of tissues targeted by SIV during acute infection.

### Cellular STAT1 RNA Levels Are Elevated During Acute SIV Infection

**Figure 6:**
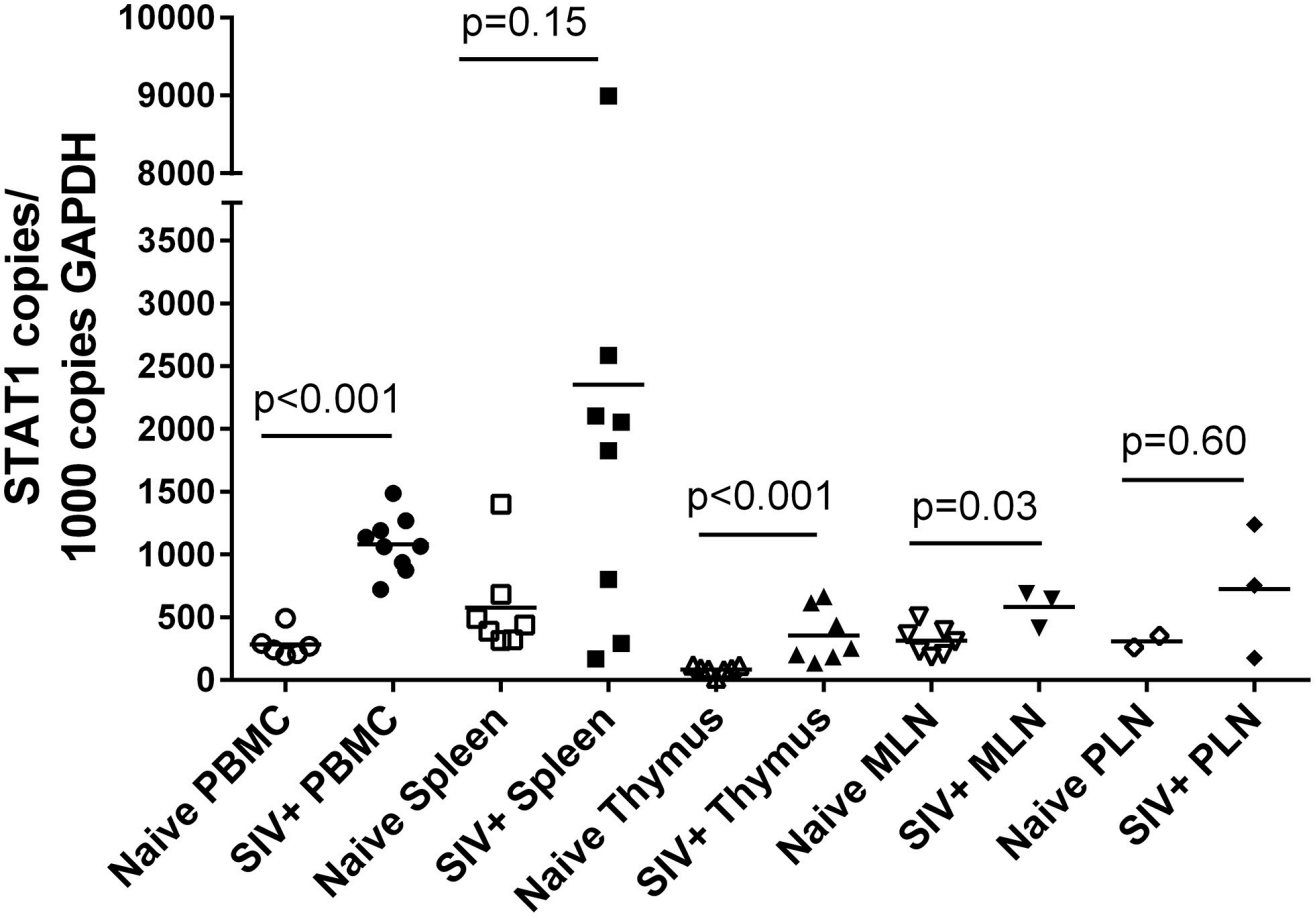
STAT1 is upregulated in acute SIV infection. Comparison of STAT1 copies in PBMC, spleen, thymus, MLN, and PLN. Open symbols represent naïve macaques and closed symbols represent SIV+ macaques. Each symbol represents the number of PcEV RNA copies from a single tissue from a single individual. Means for each group are indicated as horizontal bars with *p*-values given for a Mann-Whitney comparison of the ranked expression levels between naïve and SIV+ macaques.

STAT1 represents an independent variable in these studies and its relationship with SIV status and tissue type was determined in the same manner as for PcEV (Figure 6). As with PcEV, a global STAT1 induction was observed during acute SIV infection compared to naïve macaques. Considering each tissue separately, STAT1 was significantly increased in PBMC (Mann-Whitney test, *p* < 0.001), thymus (*p* < 0.001), and MLN (*p* = 0.03), but not spleen (*p* = 0.15) or PLN (*p* = 0.60). Hence, during acute SIV infection cellular STAT1 levels are elevated, though at variable levels for individual tissues, as SIV infection stimulates a robust immune response.

### Cell-Associated PcEV Transcription Levels Are Correlated to STAT1 in SIV+ Spleens

**Figure 7:**
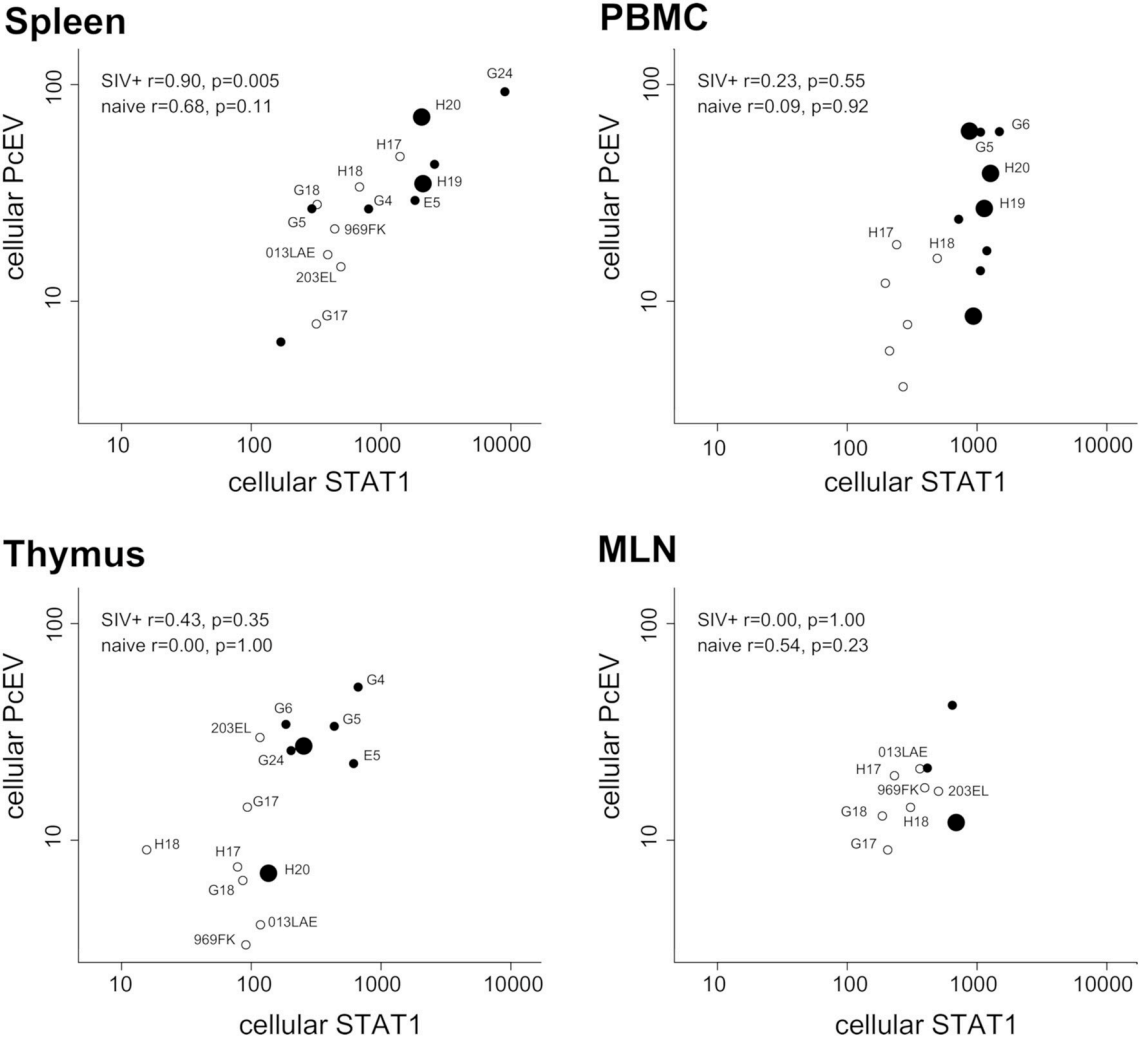
Tissue-specific relationship between cellular PcEV and cellular STAT1 RNA levels. Spleen, PBMC, thymus, and MLN compared for cellular PcEV and STAT1 RNA levels, expressed as number of RNA copies/1,000 copies GAPDH (PLN sample size was too small). For each tissue, open circles represent naïve macaques and closed circles SIV+ macaques. Large closed symbols represent SIV+ macaques in whose plasma PcEV RNA was detected. Statistical analysis of SIV+ and naïve macaques are indicated (Spearman correlation analysis). Individual animals are labeled if they appear in more than one plot.

Finally, expression levels of the two genes were compared in the same RNA preparations from each tissue sample. Figure 7 shows tissue-specific patterns identifying the relationship between localized PcEV and STAT1 expression. Cellular PcEV and STAT1 RNA levels were strongly correlated in the spleen of SIV+ macaques (Spearman test, *r* = 0.90, *p* = 0.005) and showed a positive though statistically non-significant relationship in spleens derived from naïve macaques (*r* = 0.68, *p* = 0.11). Two SIV+ macaques (H19 and H20) with elevated splenic PcEV levels were PcEV+ in plasma, but the macaque with the highest level (G24) was PcEV RNA- in plasma. Four PBMC samples from macaques that were PcEV+ in plasma (including H19 and H20) exhibited a wide range of PcEV transcription levels.

STAT1 and PcEV levels were not significantly correlated in the other tissues but most relationships were positive. Pooling all tissue data from the 22 SIV+ macaques (a total of 30 samples) yielded a strong positive correlation (Spearman test, *r* = 0.47; *p* = 0.0091). Pooled PcEV and STAT1 levels in the 13 naïve macaques were also strongly correlated (*r* = 0.61; *p* = 0.0004), albeit at a lower magnitude of induction for both PcEV and STAT1. Such correlations in pooled data might be artifactual but it is interesting that no such correlation was found between STAT1 and cellular SIV levels in these total RNA preparations (*r* = 0.29, *p* = 0.21); there was also no correlation between STAT1 and plasma SIV RNA levels (*r* = 0.25, *p* = 0.22).

## Discussion

The transcriptional activity of an ERV in macaques has been investigated using a combination of bioinformatic prediction analyses and biological measures of *in vivo* activity, exploiting archived biological materials collected from previous macaque challenge studies. PcEV expression was upregulated in several key lymphoid tissues of acutely SIV-infected macaques, when *de novo* immune responses are likely to be highest. In the spleen PcEV expression was positively correlated to cellular STAT1 RNA levels. Analysis of baseline data indicated low, variable cell-associated PcEV RNA levels in the absence of a clear external stimulus, such as SIV. Introduction of SIV induced PcEV in all tissues measured, attaining statistical significance in the thymus and PBMC. While caution needs to be exercised in not over-interpreting our findings given a number of caveats (e.g., inherent variations in SIV strain dynamics, tissues targeted during acute infection, macaque species), these data clearly suggest a global upregulation of PcEV transcriptional activity in response to acute SIV infection.

It is widely recognized that SIV establishes productive infection in multiple lymphoid tissues during the primary viraemic phase. STAT1, part of the IFN-1 cascade, represents one component of the dynamic response to SIV, triggering a range of innate immunity genes during acute infection (35), but this response fails to clear the virus in the long term. This study confirmed induction of STAT1, part of the IFN-1 cascade in response to SIV, highlighting tissue-specific differences. In the spleen, PcEV and STAT1 RNA levels were positively correlated but this relationship was less clear in PBMC, thymus and MLN and warrants further scrutiny, especially where small sample sizes precluded a comprehensive analysis. With both PcEV and STAT1, exposure to SIV *per se* appears sufficient to trigger a response independent of the level of viral replication.

Although little is known about the macaque, in the mouse model it is well-established that microbial triggering of innate and adaptive sensors leads to increased ERV transcription (51). Treatment with both lipopolysaccharide (LPS), a Toll-like receptor 4 (TLR4) agonist, and polyinosinic-polycytidylic acid [poly(I:C)], a TLR3 agonist, significantly induced expression of two mouse ERV proviruses (52). Examples of human ERVs whose expression is enhanced in the presence of IFN or viral infection include HERV-K(HML2), reported to be increased in PBMC from HIV-1-infected individuals (13, 53, 54), and HERV-W was reported to be increased by influenza virus infection *in vitro* (55, 56). The HERVK-K(HML2) LTR has two interferon-stimulated response elements (ISREs) and binding sites for inflammatory transcription factors such as NFκB (57, 58). PcEV lacks such motifs and the absence of clear STAT1 binding sites suggests that STAT1 proteins do not directly drive PcEV transcription. However, prediction of transcription binding sites is complex and lowering the dissimilarity margin of transcription binding site prediction from 5% to 15% allocates the STAT3 binding sites in PcEV also to NFκB and STAT1. STAT3 is activated at the protein level by IFN-1 (59) and binding motifs in PcEV LTR for CEPBβ, whose RNA expression is elevated by type-II IFN (60), were identified in the sequence analyses. Alternatively, PcEV might be upregulated by accessory proteins of exogenous retroviruses as HIV-1 Tat increases HERVK(HML2) transcription (61, 62). Another possibility is inflammation-induced demethylation of LTRs, where the HIV-1 5′LTR undergoes demethylation following administration of TNFα *in vitro* and LPS to transgenic mice harboring latent HIV genomes in their lymphocytes (63, 64).

With regard to how PcEV might impact on innate immune signaling pathways, two main mechanisms can be envisaged (26). The first, via an exogenous route, implies binding of ERV particles or glycoproteins to innate receptors such as TLRs, as demonstrated *in vitro* where recombinant Env glycoprotein from HERV-W triggers secretion of pro-inflammatory cytokines via TLR4 in PBMC (65). The second, via an endogenous route, is that ERV transcription and reverse transcription may generate intermediate reaction products such as dsRNA, possibly by transcription and hybridization of ERV sense and antisense transcripts, and RNA:DNA hybrids generated during reverse transcription detected by TLRs and cytosolic RIG-I-like receptors, such as MDA5 (18–20). Only low PcEV RNA levels were detected in plasma from around one-third of SIV+ macaques, suggesting PcEV particles are unlikely to be produced in large quantities. It seems plausible, however, that PcEV might trigger induction mechanisms through released viral components generated in a transient nature, supported by the finding of full-length ORFs for the *env* gene in several loci. In humans, ERV-encoded reverse transcriptase has been suggested to reverse transcribe viral RNA intracellularly (66, 67). In autoimmune TREX1-deficient mice, ERV DNA accumulates in the cytoplasm, associated with inflammatory phenotypes (23) which can be treated using RT inhibitors (68). Hence, the correlation between PcEV and STAT1 expression in this *in vivo* model requires further clarification.

One study limitation is that we restricted our analyses to data from only a single time point. This might not reveal any delayed correlations between PcEV, SIV, and STAT1 levels, especially given differences in cellular and anatomical localization of SIV (35). Cell sorting experiments to derive specific cell sub-sets would help address this issue and refine analyses. Differences in viral kinetics and tropism might also play a confounding role, although all SIV strains studied, even the minimally attenuated SIVmacC8, exhibit robust early replication kinetics *in vivo* as determined by plasma viral RNA levels 10–14 days post-infection.

To assess the relevance of the macaque as a model system, similarities and differences between human and macaque ERVs require consideration. Humans have a single ERV lineage that has been replicating within the last few million years: HERV-K (HML2). Early studies reported HERV-K(HML2) RNA in plasma from HIV patients (53), but more recent studies failed to reproduce this (13, 14). Evidence for low level PcEV RNA was identified in the plasma of a proportion of SIV+ macaques. Detection of retroviral RNA in plasma presumes some form of protein protection, whether as fully or partially packaged particles, so some macaques in the population may harbor a replication-competent PcEV locus. Although we did not find any potentially replication-competent PcEV in the reference macaque genome sequences, this may reflect a combination of genome assembly problems with the likelihood that such loci would be unfixed in the macaque population, i.e., present only in the genomes of some individual macaques (42). Evidence for at least two unfixed PcEV loci was found. This phenomenon occurs in HERV-K(HML2), where loci with full-length ORFs in all genes are present only in some non-reference genomes (69, 70). Replication-competent PcEV loci could also be generated by recombination: in the mouse model, recombination between replication-defective loci reconstituted replication-competent loci with resulting infectious virions (71). Although macaques might differ from humans in having as yet unrecovered replication-competent PcEV loci, levels of cell-associated HERV-K(HML2) transcription appear similar: ~30 copies/1,000 copies GAPDH in PBMC of non-acute HIV infection cases and ~15 RNA/1,000 copies GAPDH in PBMC from uninfected individuals (13).

Assuming SIV induces PcEV expression *in vitro*, knock-down of PcEV transcription and individual sensors followed by measurement of the IFN-1 response would determine whether PcEV is involved in the IFN-1 response. The other two recently active ERV lineages in the macaque, Chimpanzee Endogenous Retrovirus (CERV), and Simian Endogenous type D Retrovirus (SERV) (25), also require study since within both lineages single examples of loci with full-length ORFs in all genes were identified. Another important group of retroelements, Long Interspersed Nuclear Elements (LINEs), also require scrutiny because they, unlike ERVs, are known to be copying in the human population (72) and potentially replication-competent loci have been found in the macaque genome (73). LINEs have an entirely intra-cellular replication cycle that would help is distinguishing between endogenous and exogenous routes of innate triggering. Considering several ERVs and LINEs together would provide a wider perspective on whether retroelements are involved in fundamental immune response induction.

In conclusion, the current study demonstrates that PcEV retains transcriptional activity despite the apparent lack of replication. Expression appears to be upregulated by acute SIV infection and in some tissues a correlation exists between the expression levels of PcEV and STAT1, the latter being part of the IFN-1 cascade. The mechanistic processes at play warrant detailed investigation to determine whether ERVs are overlooked components of innate immune responses and activation signaling mechanisms.

## Materials and Methods

### Extraction and Alignment of PcEV Locus Sequences

Bioinformatic analyses were performed using UCSC's Genome Browser website and its BLAT tool (74) on the most recent reference genome sequence of the RM, rheMac8 (Mmul_8.0.1). This assembly derives from short-read Illumina whole genome sequencing (MacaM) (75) with later closing of some gaps using long-read PacBio sequencing of the same individual animal (comment in GenBank accession GCA_000772875.3).

Sequences of loci were compared with those from an earlier build of the RM genome, rheMac2 (76) (GenBank accession GCA_000002255.2). This build is an earlier short-read whole genome sequencing project largely from the same (female) individual (ID 17573), with some sequencing of an unrelated male used in finishing and to provide a Y chromosome. Sequences were also compared to those of a CM, a closely related species sharing a common ancestor only 0.91 (±0.11) million years ago, which is more recent than the common ancestor of all rhesus macaques (38). The reference genome sequence of the CM, macFas5, appears to have been built independently of the RM genome using a range of short-read technologies (comment in GenBank accession GCA_000364345.1). The other RM genome assembly available on the UCSC website, rheMac3, is very fragmentary.

PcEV was described initially from the baboon (24) (GenBank reference AF142988), erroneously referred to as BaEV in an earlier analysis of the macaque genome (25) (see Supplementary Information). This GenBank sequence was used to locate and download the sequences of several PcEV loci in the macaque using the UCSC Genome Browser. A multiple alignment was then made of the downloaded sequences using MEGA (77, 78) and a new consensus reference sequence built with full-length ORFs in all genes (sequences from different loci differed only by a few percent so alignments were unambiguous). This reference was used to re-search the macaque genome. Multiple alignments were made manually from downloaded sequences and individual loci examined visually for full-length ORFs and other motifs.

Previously published data on recently active macaque ERVs lineages (11, 25) are presented here in more detail (Figures S2–S4).

### Prediction of Transcription Factor Motifs

A consensus PcEV LTR sequence was built based on an alignment of LTR sequences from the loci displayed in Figure 1, excluding the two much older loci, plus NC_022517 (36) and AF142988 (24). This sequence was screened with the ALGGEN-PROMO online tool (79, 80) for the presence of putative binding sites for a range of transcription factors linked to either inflammation or retroviral infection (43, 44). The dissimilarity between query and binding site matrices from the database was fixed at a threshold of 5%. The following transcription factors and binding sites were selected for NHPs only, with the accession number in the TRANSFAC public database given in square brackets: AP1 [T00029]; NFI/CTF [T00094]; C/EBPα [T00105]; NFY [T00150]; CREB [T00163]; cRel [T00168]; CTF [T00174]; GATA1 [T00306]; GATA2 [T00308]; GATA3[T00311]; GRα[T00337]; NF1[T00539]; NFAT1[T00550]; C/EBPβ[T00581]; C/EBPΔ[T00583]; NFkB[T00590]; NFkB1[T00593]; RelA [T00594]; POU2F1 [T00641]; Sp1 [T00759]; TBP [T00794]; YY1 [T00915]; STAT1α, [T01492]; STAT3 [T01493]; STAT1β [T01573]; NFYA [T01804]; GRβ [T01920]; NFAT2 [T01945]; NFAT1 [T01948]; STAT1 [T04759]; GR [T05076].

### Dating the Integration of ERV Loci

Integrations were dated using the nucleotide divergence between the two LTRs of the proviruses. These LTRs form the flanks of the provirus (the complete integrated DNA form of a retrovirus) and are identical at the time of integration, subsequently accumulating substitutions at the host background rate. The following equation was used to calculate locus age (81): integration time = sequence distance between 5′ and 3′ LTR/(rate in 5′ LTR + rate in 3′ LTR).

Thus, estimated age = (a/b)/r^*^2 where,

a = number of mismatches between the two LTRs,

b = length of the LTR,

*r* = estimated rate of nucleotide substitution.

Note, because substitutions will have occurred along the two branches leading to the 5′ and the 3′ LTRs it is possible to alternatively multiply the LTR length by two to reflect the effect of this. The rate of nucleotide substitution was taken as 1.0 × 10^−9^substitutions/nucleotide/year based on analyses of primate ERV sequences (25). This value is about half the rate estimated for mammals generally (82, 83) but is close to estimates for the rate of neutral molecular evolution in Old Word Monkeys and Apes (38).

For loci with no substitutions within their LTRs, the probability (*P*_0_) of observing this if the locus had integrated at time “age” can be found from the Poisson distribution with *P*_0_ = e^−λ^ where λ is the average number of events in the time period. Thus, using the notation *P*_0_ = e^(−2*b*r*age)^, the probability of having no substitutions in the LTRs is just <0.5 at age 700,000 years.

### Study Population and RNA Preparation

Plasma and cells were derived from acutely SIV-infected Indian RM and CM challenged with different SIV isolates in historical studies of SIV pathogenesis and vaccination (35, 48, 50, 84). Total RNA was extracted from 140 μL to 1 mL plasmas using QiaAmp (Qiagen Ltd) according to the manufacturer's instruction. Plasmas obtained from CM were extracted from a volume of 140 uL, the four RM plasmas were extracted from 1 mL plasma. Each RNA preparation underwent a DNase step to remove contaminant DNA and all qPCR samples were run with an RT minus control, an essential step when working with ERVs. Healthy human plasma contains about 1,000 genome copies/mL, in the form of cell-free DNA (85), with many ERV copies in every cell. By contrast, exogenous retroviruses have only a single DNA copy in a small minority of PBMC (86), so the danger of DNA contamination is greatly reduced. For quantification of cell-associated transcripts, RNA was extracted from frozen cells, washed once in PBS and lysed with guanidine isothiocyanate (Sigma Ltd). Chloroform (200 μL) was added followed by centrifugation at 13,000 rpm for 2 min, aqueous phase collected, 1 volume 100% ethanol added and samples loaded onto an RNEasy silica column (Qiagen Ltd). Columns were washed and RNA eluted according to the manufacturer's instruction to a final volume of 50 μL and quantitated using nanodrop. Each RNA preparation was diluted to 10 ng/μL, and 50 ng (5 μL) added to each qPCR reaction.

### Primers and Probe Sequences

Primer sequences for qPCR are shown below in 5′-3′ orientation. PcEV primers were based on an alignment of PcEV *pro-pol* sequences (Figure S5) designed to anneal to all loci. SIV primers are based on conserved regions in *gag* (49) except for SIVsmE660 based on conserved LTR sequences (50). PcEV, GAPDH, and STAT1 sequences were as follows:

PcEV: CCGTGTCTATCAAGCAATATCC (forward), GGCAGAAGAGGAGTGTTCCAGG (reverse) and AACTCGGAGTGTTGCGAC (probe).

GAPDH: GGCTGAGAACGGGAAGCTC (forward), AGGGATCTCGCTCCTGGAA (reverse), and TCATCAATGGAAGCCCCATCACCA (probe).

STAT1: CAATACCTCGCACAGTGGTTAGAAAA (forward) and CGGATGGTGGCAAATGAAAC (reverse).

SIVsmE660: CTCCACGCTTGCTTGCTTAA (forward), AGGGTCCTAACAGACCAGGG (reverse), TCCCATATCTCTCCTAGYCGCCGC (probe).

Other SIVs: AGTGCCAACAGGCTCAGAAAA (forward), TGCGTGAATGCACCAGATG (reverse) and TTAAAAAGCCTTTATAATACTGTCTGCG (probe).

### Plasmids and *in vitro* Transcription

pBluescript II SK+ (pBS, 3.0 kb) was from Stratagene Ltd (1 μg/μL). pBS-PcEV was derived separately by inserting a PcEV amplicon into the multiple cloning site of a pBS vector using *Apa*I (nt 604) restriction sites (GENEWIZ, Inc). pBS-PcEV was linearized by *Hind*III restriction located 16nt downstream of the PcEV sequence. Reaction mixes included 1 μg of pBS-PcEV plasmid (0.2 μg/μL), 2 U/μL final concentration of enzyme and 15 μL of water/enzyme buffer. Linearization was at 37°C for 1 h. *In vitro* transcription and purification were carried using MAXIscript kit (Ambion Ltd) following manufacturer's instructions.

### Standard Curves

PcEV transcripts were DNase-treated and 10-fold serial dilutions made. PcEV copy number was determined by Poisson end-point calculations, with Ct values obtained for each plasma sample compared to the standard curve to estimate number of PcEV copies per reaction (for 5 μL total RNA input). The number of PcEV copies/mL for each plasma sample was determined taking into account extraction starting volume. SIV standards used were derived from a SIVmac251 plasma RNA reference series (49, 87). For intracellular RT-PCR assessments, standards for GAPDH and STAT1 were as previously described (35).

### DNase Treatment of RNA Preparations

Removal of DNA contaminant was conducted using DNA-free kit (Ambion Ltd). 5 μL of 10X DNAse buffer was added to 50 μL RNA preparation. 2 μL TURBO DNase was added and incubated for 1 h at 37°C. 10 μL Inactivation Reagents were added and incubated for 5 min with intermittent flicking of tubes. Inactivation reagents were pelleted by centrifugation at 10,000 g for 2 min. Supernatant containing RNA was harvested, assayed or stored at −80°C for further analysis. In the absence of DNAase treatment, similar copy numbers between RT+/RT- reactions were noted: H17(RT+ = 1.18E+04; RT− = 1.27E+04); H18(RT+ = 7.06E+03; RT− = 9.12E+03); H19(RT+ = 2.39E+04; RT− = 2.37E+04); H20(RT+ = 6.96E+03; RT− = 8.99E+03). Observed levels of DNA contamination are consistent with theoretical expectations: assuming macaques have similar levels of cell-free DNA in plasma, given at least 50 intact PcEV copies exist in each macaque cell, with an expectation of at least 5 × 10^4^ DNA copies in plasma.

### qPCR and qRT-PCR

Quantification of PcEV and SIV transcripts in plasma were determined with 5 μL of DNase-treated RNA per qPCR reaction, assayed in triplicate using RNA Ultrasense One-Step Quantitative RT-PCR kit (Invitrogen Ltd). For PcEV, qPCR reactions consisted of 25.9 μL nuclease-free water, 1 μL forward and reverse primers (5 μM each, final concentration: 100 nM), 2.5 μL PcEV-specific Taqman probe (10 μM), 10 μL of 5X master mix, 0.1 μL ROX reference dye, 2.5 μL Superscript III RT and Platinum Taq polymerase enzyme mix. Cycling parameters were 48°C for 30 min RT step, 95°C for 5 min, 95°C for 15 s (40 cycles); 61°C extension 1 min. For SIV, mix contained 25.9 μL nuclease-free water, 1 μL each forward and reverse primer (5 μM each, final concentration: 100 nM), 2.5 μL SIV-specific probe (10 μM). Cycling for 52°C 60 min RT step, 95°C 10 min, 95°C for 30 s (40 cycles) and 61°C extension for 90 s.

For quantification of cell-associated transcripts, each RNA preparation was diluted to 10 ng/μL, so that 50 ng (5 μL) was added in each qPCR reaction. For PcEV and SIV, qPCR was conducted as stated above. For GAPDH, the mix contained 25.9 μL nuclease-free water, 1 μL each forward and reverse primer (5 μM each, final concentration: 100 nM), 2.5 μL GAPDH-specific probe (10 μM). Cycling parameters were 52°C 60 min RT step, 95°C, 10 min, 40 cycles of 95°C, 30 s (40 cycles) and 61°C extension for 90 s. For STAT1, the Power SYBR Green RNA-to-Ct 1-step kit was used (Applied Biosystems Ltd). Mix consisted of 15.6 μL nuclease-free water, 4 μL each forward and reverse primer (10 μM each, final concentration: 400 nM), 25 μL 2X Master Mix (containing SYBR Green and polymerase) and 0.4 μL RT-enzyme mix. Cycling parameters were 48°C 30 min RT, 95°C for 10 min, 95°C for 15 s (40 cycles) and 60°C extension for 1 min. Melt curve was 95°C for 15 s, 60°C for 15 s and 95°C for 15 s. RNA preparations were confirmed to lack detectable DNA by performing a parallel quantitative PCR lacking reverse transcriptase for PcEV, SIV and GAPDH on all RNA extracts. In the RNA Ultrasense system, enzyme mix was replaced by 0.2 μL Platinum Taq polymerase (10 U/μL) (Invitrogen Ltd).

### Statistical Analysis

GraphPad Prism version 6.01 was used for all statistical analyses. The Mann-Whitney test was performed for comparing non-paired samples in naïve vs. SIV+ groups; the Kruskal-Wallis test was used to compare expression levels between macaques infected with different strains of SIV. Since most datasets had distributions that were significantly different from normal, correlation coefficient *r* and *p*-values are presented from a non-parametric Spearman test. Fisher's Exact Test was used to assess whether SIV is associated with PcEV in the plasma of SIV+ animals.

## Ethics Statement

All biological samples used in this study were derived from previously conducted experiments, which received the appropriate ethical approval from the local NIBSC ethical committee. Animal procedures were performed in strict accordance with UK Home Office guidelines under a license granted by the Secretary of State for the Home Office which approved the work described. These previous experiments have been published (35, 48, 81–83, 85) along with full details of experimental design, procedures and ethical approval.

## Author Contributions

EM, NB, and RB conceived and designed the experiments. EM and CH performed the experiments. RB, JK, and LU performed the bioinformatic analyses. EM, NA, GT, NB, and RB wrote the paper. EM and RB carried out the statistical analyses. All authors approved the final draft of the paper.

### Conflict of Interest Statement

The authors declare that the research was conducted in the absence of any commercial or financial relationships that could be construed as a potential conflict of interest.
