# Supplementary material for: Variable Baseline Papio cynocephalus Endogenous Retrovirus (PcEV) Expression Is Upregulated in Acutely SIV-Infected Macaques and Correlated to STAT1 Expression in the Spleen
Source: Front Immunol. 2019 May 15;10:901. doi: 10.3389/fimmu.2019.00901 (PMC6529565; doi:10.3389/fimmu.2019.00901)
Supplement: Supplementary file 1 [file Data_Sheet_1.PDF]

Supplementary Information
Variable baseline *Papio cynocephalus* Endogenous Retrovirus (PcEV) expression is upregulated in acutely SIV-infected macaques and correlated to STAT1 expression in the spleen

Emmanuel Atangana Maze; Claire Ham; Jack Kelly; Lindsay Ussher; Neil Almond, Greg Towers, Neil Berry and Robert Belshaw.

Number of words excluding figure and table captions: 1447
Number of supplementary figures: 5
Number of supplementary tables: 1

1 SUPPLEMENTARY DATA

1.1 Supplementary Results

Details of analysed PcEV loci

- **Chr1:55452680-60247** (+ve sense). This represents one of only two loci with identical LTRs (see also chr5:114918771-21894 below), suggesting integration at least within the last 700,000 years (see Methods). Consistent with being a relatively recent integration, this locus also appears to be heterozygous: an identical match to the reconstructed pre-integration site – extending 100nts either side of the Target Site Duplication (TSD) – was identified in an unassembled part of the same genome (chrUn_NW_014907418v1), and the earlier rheMac2 assembly from the same animal contains only an identical match to the same reconstructed pre-integration site and not to the provirus. Confirming homology at this locus is slightly complicated because the locus is within an older ERV integration (designated HERV-17-int in RepeatMasker), and thus the flanking regions will themselves have homology to other regions in the genome. Fortunately, this older ERV locus into which PcEv has integrated has diverged markedly from other members of the same HERV-17-int family such that the second best match is only 91% similar. This provides confidence that the same integration event occurs in different builds and in different animals (see file PcEV_data.txt for the flanking sequences). Unfortunately, the internal region of this locus is both incomplete and possibly even comes from more than one locus: *gag* is highly degenerate with many frameshifting indels while the (incomplete) *pro-pol* has a potential full-length ORF.
- **Chr1:121693612-70205** (-ve sense). *Gag* and *pro-pol* sequences are identical to those in rheMac2. The earlier rhesus build also shares the16 amino acid insertion near the start of *env* (as does the cynomolgus genome) and has the same four mismatches between the two LTRs. As expected from their estimated age, this and the other intact loci in the rhesus were all also found in the CM genome.
- **Chr1:223875631-67155** (-ve sense). A full-length locus was identified in the rhe2Mac2 build, except the latter is +ve sense. This allowed

confirmation of all premature stop codons and frameshifting indels in Figure 1 and correction of some sequencing errors, *e.g.* in *pro-pol* a frameshifting loss of one nucleotide from a run of three Gs in rheMac8 does not occur in rheMac2.

- **Chr2: 120398819-411598** (-ve sense). *Gag* in rheMac8 has many frameshifting indels, none of which are in rheMac2, and the one stop codon in rheMac2 is not in rheMac8 (this sequence does not appear to be in the CM genome). The *pro-pol* gene has multiple assembly errors, leading to both builds appearing to have long tandem duplications, but neither of these tandem duplications appear in both builds (and neither do any of the premature stop codons or other frameshifting indels); however, the premature stop codon in the centre of the rheMac2 *pro-pol* is also in the CM genome, indicating that it is genuine.
- **Chr3:28091955-100392** (-ve sense). The first are 47nts are missing from *gag* in both RM builds but the ORF is intact in CM. Other indels are found only in one of the two rhesus builds.
- **Chr3:28106593-15637** (+ve sense). Contains the only full-length *pro-pol* ORF recovered, although there is a frameshifting indel near the end of *gag* that would throw *pro-pol* out of frame. Scaffold gaps are not repeated in both builds.
- **Chr5: 114918771-21894** (+ve sense). As with chr1:55452680-60247 (above), this locus has identical LTRs. This locus spans a gap in the scaffold of the rheMac8 build and has little of the internal coding region (<2,000nts). Also similar to the situation with chr1:55452680-60247, the locus is within an older ERV locus (designated LTR35B in RepeatMasker) but detection of the pre-integration site in the rheMac3 genome sequence can be made with similar confidence: a 400nt region at very close genomic coordinates contains only two substitutions compared to rheMac8 (99.5% similarity) while the next best match elsewhere in the rheMac3 genome is 78.6%.
- **Chr5:123585628-593787** (-ve sense) Gaps are not repeated in both rhesus builds.
- **Chr8:14596826-605415** (+ve sense). An old but complete locus with many premature stop codons (note, the alignments in Figure 1 are in frame).
- **Chr9:50301699-10531** (+ve sense). This locus is discussed in the main text and the analysis shown in Figure 2. The locus was located in different genomes by searching for unique chimaeric sequences consisting of the end of the LTR and the contiguous host flanking region. Coordinates of this locus in the rheMac2 build were chr9:56546894-55724 and in the macFas5 build of the CM were chr9:55063875-78763. It is only the upstream part of *pro-pol* in rheMac8 that is a problem: both (a) the region containing the 5' LTR plus the start of *gag* and (b) *env* plus the 3' LTR were very similar in the three builds. In addition, the macFas5 build has a break in the scaffold near the end of *pro-pol* (which appears to involve some sequence duplication). In the reconstruction of this locus shown in Figure 2, we chose not to infer a second premature stop codon and a frame-shifting indel in the homologous region of *pro-pol,* although these are present in both rheMac2 and macFas5. It is unclear why these substitutions are not also in the *pro-pol* region of rheMac8 that our

phylogenetic analysis suggests is homologous. A more detailed analysis of ERVs the macaque genome sequences is required to resolve this. The 2 LTRs of this locus in rheMac8 differ by a total of 4 substitutions. In the earlier build these differed by 3 substitutions, only 2 of which are shared in both builds.

- **ChrX:88958264-866804** (-ve sense). This old locus was not identified in the rheMac2 build, although it is present in macFas5.
- **ChrY:10651278-9714** (-ve sense). The rheMac2 build and the cynomolgus genome are both from females hence it was not possible to check the rheMac8 sequences of this locus (although the rheMac8 build is from a female it includes the Y chromosome from another individual).

1.2 Supplementary Materials and Methods

Overview of the macaque genome

Our reanalysis of the data presented in ref 1 show the 3 ERV lineage that have been copying within the macaque genome in the last 5 million to have similar levels of recent integration (Figure S4). In this earlier study, PcEV was erroneously referred to as BaEV (Baboon Endogenous Retrovirus). BaEV appears to have been formed in the baboon lineage by a recombination event between PcEV and another ERV, SERV, and was first described from several baboon species (2). BaEV contains the *gag* and *pro-pol* genes of PcEV and the *env* of SERV (3). BaEV was confirmed to be absent from the macaque by BLATing the complete BaEV reference sequence (NC_022517) to the macaque genome sequence. The top matches were to PcEV loci shown in Figure 1 but only the first ~5000nts of the 8507nts were matched. BLATing the unmatched 3' region recovered loci that were identified as SERV. Thus, it is possible to conclude that while the baboon has both PcEV and BaEV lineages, only the former is present in the macaque.

In humans, the most recently active ERV lineage is HERVK, or more accurately HERVK(HML2), which appears to have been copying in humans at least until 250,000 years ago (4). However, the sister lineage of HERVK in the macaque ceased copying ~5mya (1) and in our study the loci appeared to be much more degraded than either HERVK in humans or PcEV in the macaque, since only 2 full-length ORFs were identified.

Figure S3 illustrates the 3 ERV lineages (plus HERVK) among a dendrogram of all macaque ERV loci that integrated roughly since the platyrrhine/catarrhine split. Their relative youth is shown by the number of short branches near the tip (bottom) of the tree. As mentioned in the Introduction, larger-bodied animals tend to have had fewer ERV integrations within the last 10mya (5) and in that study the macaque is intermediate between the mouse and humans. Indeed, while the macaque and humans are on the regression line, the mouse has even more recently integrated loci than predicted for its small size. This can be further supported by comparing dendrograms of the mouse, macaque and human (Figure S2). The macaque thus appears to offer a model system closer to the human than the mouse in this respect.

Note on rheMac8 genome assembly

RM genome assembly rheMac8 (Mmul_8.0.1) is from same (female) animal as rheMac2, namely animal 17573 (6). This papers states that the bulk of the sequencing was from a female but an unrelated male was used for the BAC-end sequencing and

to 'aid in selective finishing'. It is assumed this individual provided the Y chromosome.

2 SUPPLEMENTARY FIGURES AND TABLES

2.1 Supplementary Figures

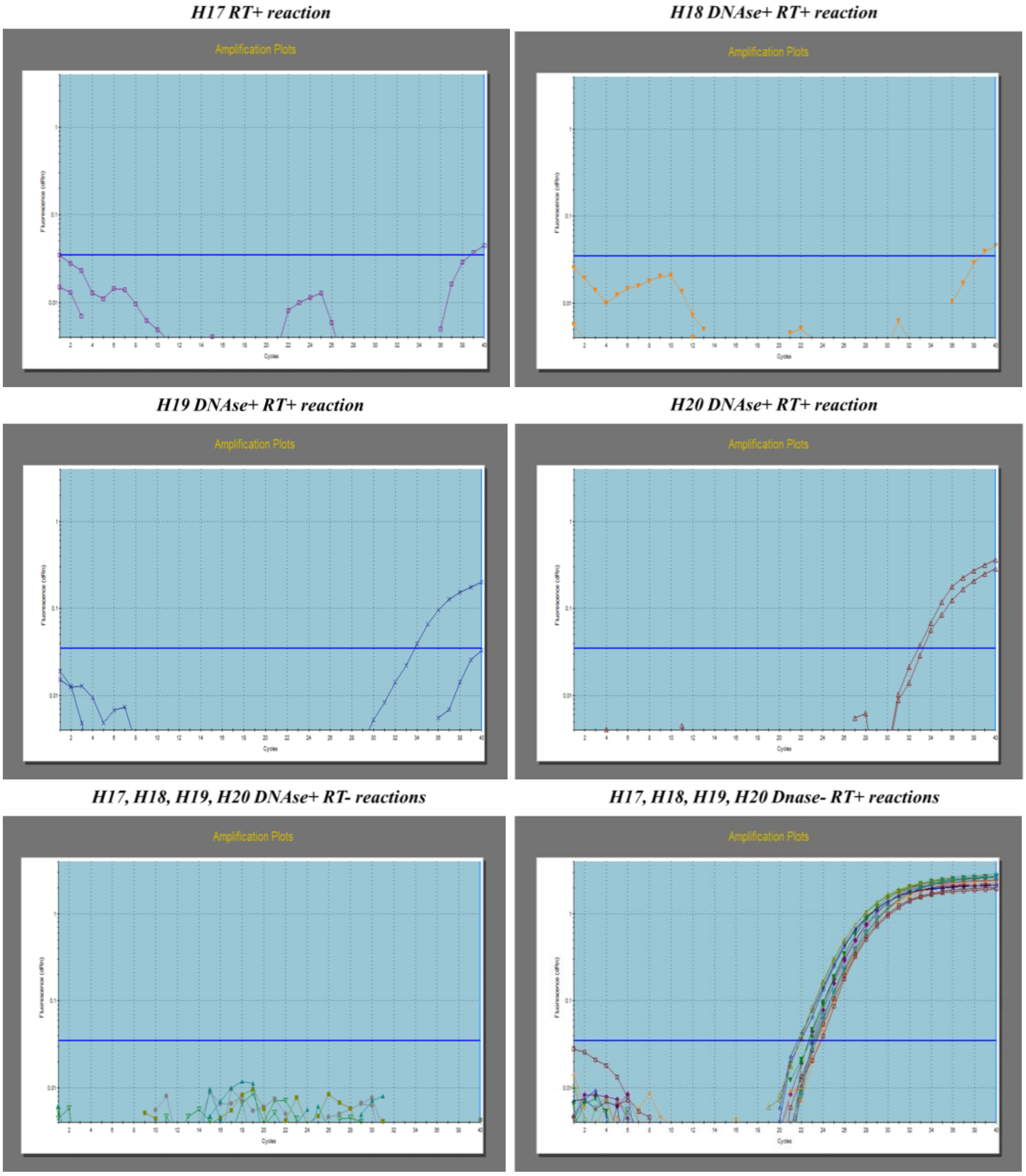

Figure S1. Amplification plots for PcEV duplicate reactions +/- RT and +/- DNase treatment. H17, H18, H19 and H20 from table 1 in the main text are displayed.

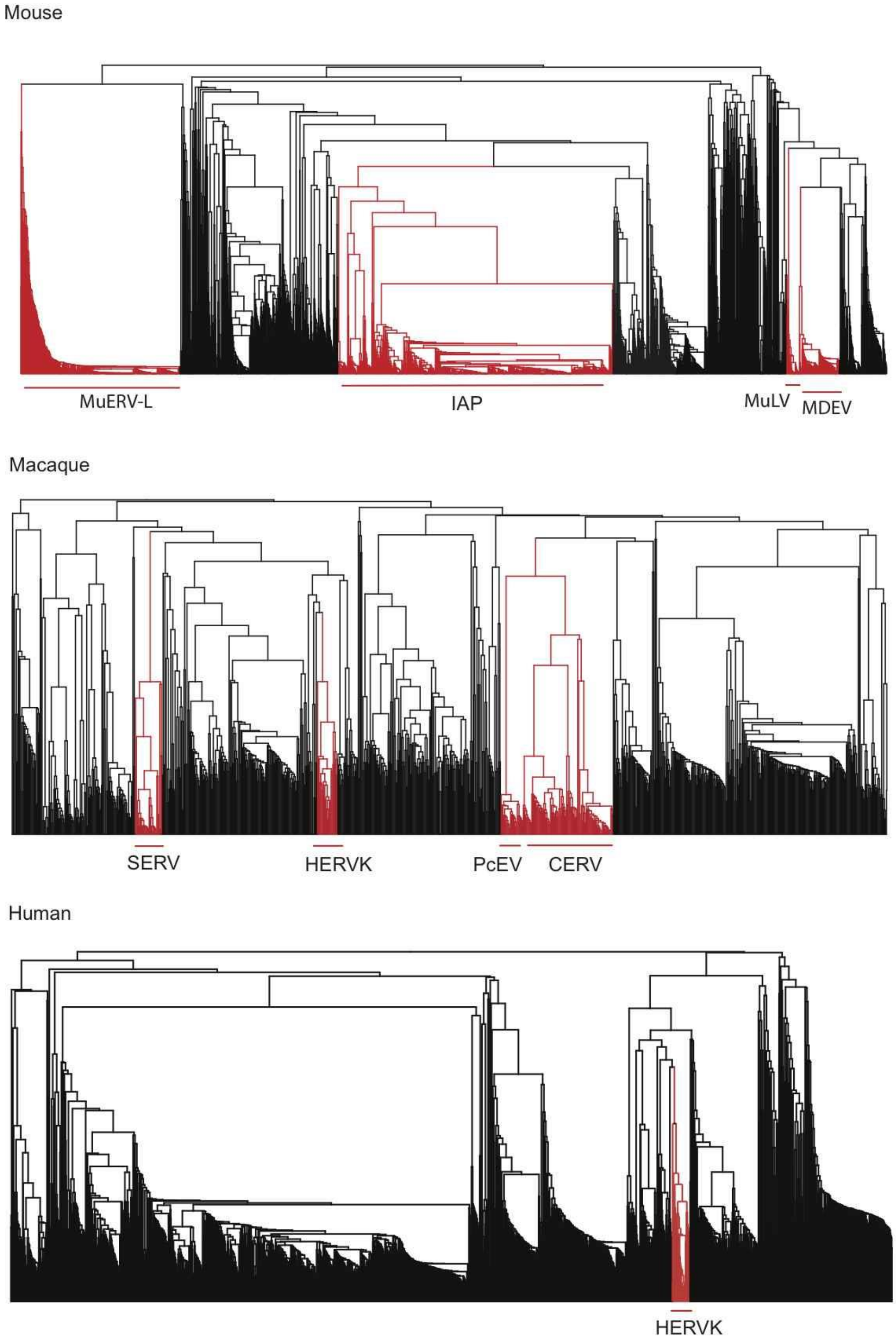

Figure S2. Dendrogram comparing relative abundance of ERV lineages, shown in red, that contain loci integrating within the last few million years, in the mouse, macaque and human genomes. Details of how the dendrogram were built and their interpretation are given in the legend of figure S3. Dendrograms for human and macaque are taken from ref 1; mouse dendrogram built using an identical method to the one used in that study with data from ref 8.

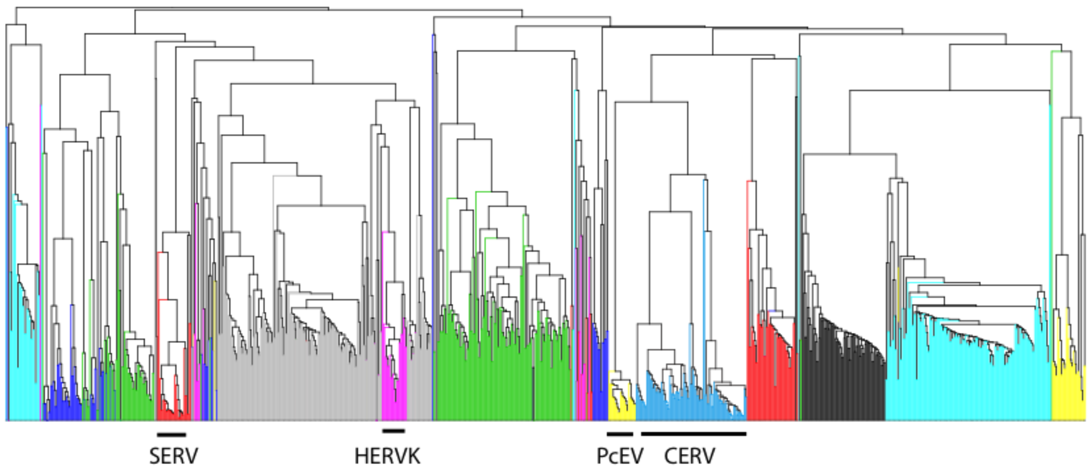

Figure S3. Dendrogram showing abundance of recently integrated ERV loci in mouse compared to rhesus macaque and human genomes, modified from ref 1. UPGMA dendrogram using pairwise similarity of *pol* gene with very old (>35my) loci excluded. The pair-wise dissimilarity matrix included all loci having at least a 300nt long *pol* match in a region of at least 90% sequence identity with at least one other locus (thus removing loci that would have integrated before the platyrrhine/catarrhine split). The 4 most recently integrated ERV lineages in the macaque are labelled. SERV appears in the class II clade (related to alpha and beta exogenous viruses) and a sister group relationship exists between PcEV and CERV in the class I clade (related to gamma exogenous viruses). CERV shows a long relationship with the macaque genome and probably multiple invasions (there appear to have been multiple invasions of this ERV in the Great Apes (7)).

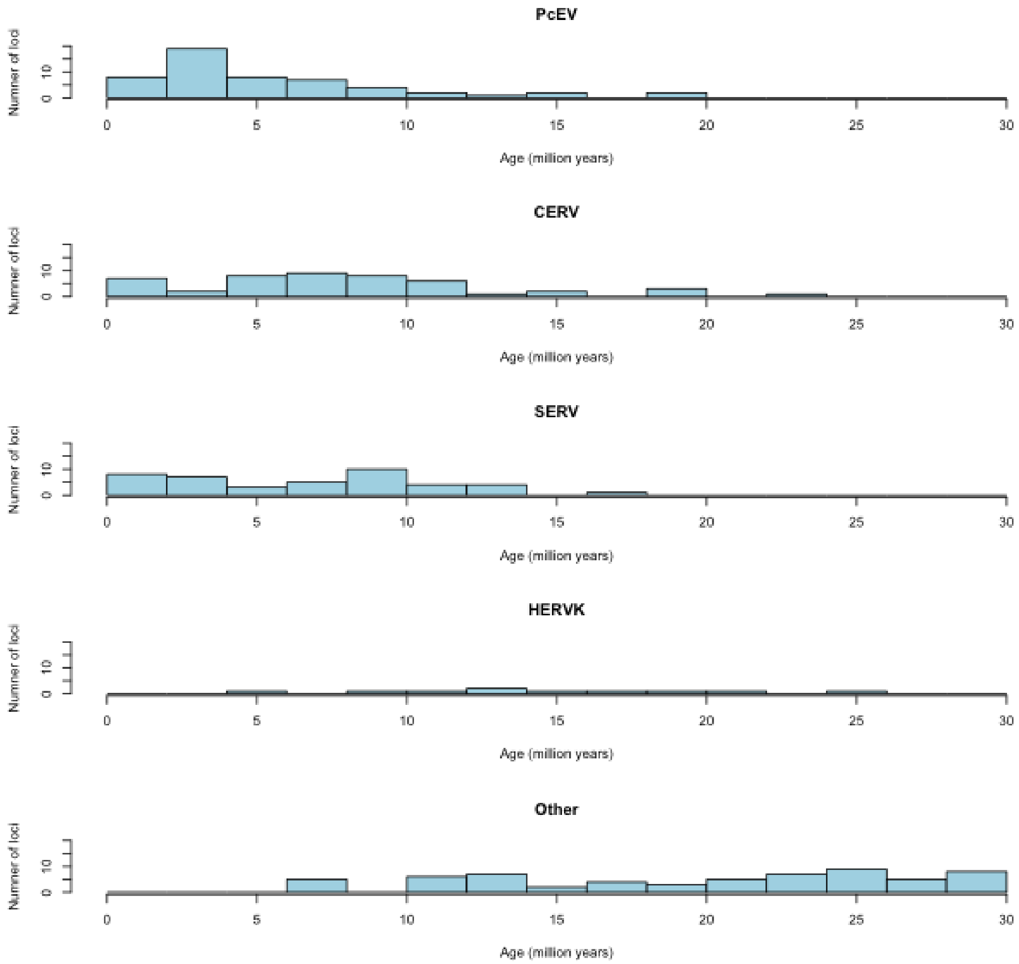

Figure S4. Histogram showing number of ERV integrations in the rhesus macaque genome (build rheMac2) in 2 million year periods, with integrations dated using LTR divergence. Data re-analysed from ref 1.

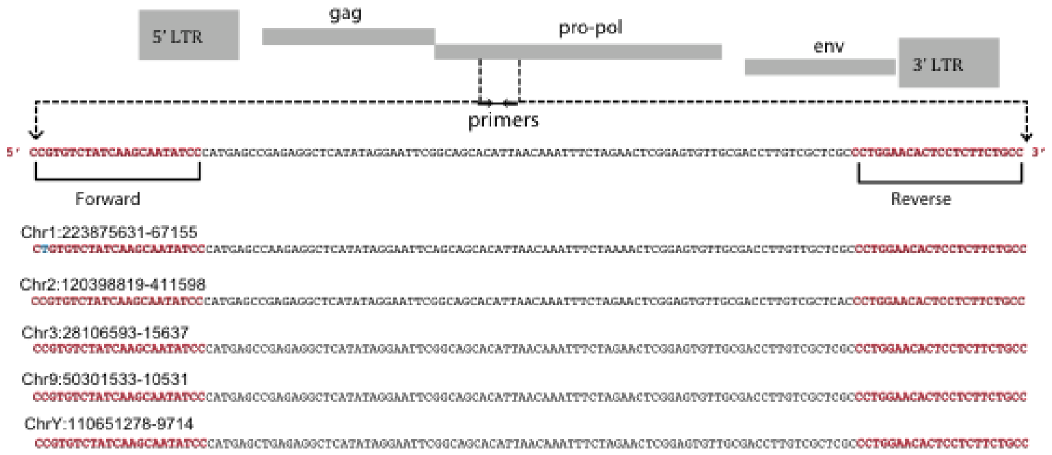

Figure S5. Conservation of primer sites in the most intact loci, which are shown in Figure 1. Substitutions within primer sites are shown in blue.

2.2 Supplementary Tables

Table S1. Summary of transcription factor binding domains. 'Yes' means no mutation in the core TATA box (TATAAA), and hence at least some transcription. In the other columns, '+' means no mutation and '-' means at least one mutation in the DR core (GATAGGG) or the CCAAT box core (CCAAT). The number of '+'s in each row is summed in the right hand column to give an estimate of the potential strength of transcription.

	TATAAA	DR1	DR2	DR3	CCAAT box 1	CCAAT box 2	CCAAT box 3	CCAAT box 4	Potential strengh
Chr1:121693612-121702053 (-ve sense)	Yes	-	-	-	-	-	+	+	++
Chr1:223875631-67155 (-ve sense)	Yes	+	+	+	+	+	+	+	+++++++
Chr2:120398819-411598 (-ve sense)	Yes	-	-	+	+	-	+	+	++++
Chr3:28091955-100392 (-ve sense)	Yes	-	+	+	+	+	+	+	++++++
Chr3: 28106593-15637 (+ve sense)	Yes	+	+	+	+	+	+	+	+++++++
Chr9:50301533-10531 (+ve sense)	Yes	-	-	-	-	-	+	+	++
ChrY:110651278-9714 (-ve sense)	Yes	+	+	+	+	+	+	+	+++++++
Chr1:55452680-60247 (+ve sense)	Yes	+	+	-	+	+	+	+	++++++

SUPPLEMENTARY REFERENCES

1. Magiorkinis G, Blanco-Melo D, Belshaw R. The decline of human endogenous retroviruses: Extinction and survival. *Retrovirology* (2015) **12**:8. doi:10.1186/s12977-015-0136-x
2. van der Kuyl AC, Dekker JT, Goudsmit J. Full-length proviruses of baboon endogenous virus (BaEV) and dispersed BaEV reverse transcriptase retroelements in the genome of baboon species. *J Virol* (1995) **69**:5917–24.
3. van der Kuyl AC, Mang R, Dekker JT, Goudsmit J. Complete nucleotide sequence of simian endogenous type D retrovirus with intact genome organization: evidence for ancestry to simian retrovirus and baboon endogenous virus. *J Virol* (1997) **71**:3666–76.
4. Marchi E, Kanapin A, Magiorkinis G, Belshaw R. Unfixed Endogenous Retroviral Insertions in the Human Population. *J Virol* (2014) **88**:9529–9537. doi:10.1128/JVI.00919-14
5. Katzourakis A, Magiorkinis G, Lim AG, Gupta S, Belshaw R, Gifford R. Larger Mammalian Body Size Leads to Lower Retroviral Activity. *PLoS Pathog* (2014) **10**:e1004214. doi:10.1371/journal.ppat.1004214
6. Zimin A V., Cornish AS, Maudhoo MD, Gibbs RM, Zhang X, Pandey S, Meehan DT, Wipfler K, Bosinger SE, Johnson ZP, et al. A new rhesus macaque assembly and annotation for next-generation sequencing analyses. *Biol Direct* (2014) **9**:20. doi:10.1186/1745-6150-9-20
7. Yohn CT, Jiang Z, McGrath SD, Hayden KE, Khaitovich P, Johnson ME, Eichler MY, McPherson JD, Zhao S, Pääbo S, et al. Lineage-specific expansions of retroviral insertions within the genomes of African great apes but not humans and orangutans. *PLoS Biol* (2005) **3**:e110. doi:10.1371/journal.pbio.0030110
8. Magiorkinis G, Gifford RJ, Katzourakis A, De Ranter J, Belshaw R. Env-less endogenous retroviruses are genomic superspreaders. *Proc Natl Acad Sci* (2012) **109**:7385–7390. doi:10.1073/pnas.1200913109
